# *Potentilla anserina* L. developmental changes affect the rhizosphere prokaryotic community

**DOI:** 10.1038/s41598-021-82610-9

**Published:** 2021-02-02

**Authors:** Yaqiong Wang, Yuxi Liu, Xue Li, Xiaoyan Han, Zhen Zhang, Xiaoling Ma, Junqiao Li

**Affiliations:** 1 School of Ecology, Environment and Resources, Qinghai Nationalities University, Bayi Road, Xining, 810007 Qinghai China; 2 Qinghai Provincial Key Laboratory of High-Value Utilization of Characteristic Economic Plants, Xining, 810007 China; 3 Qinghai Provincial Biotechnology and Analytical Test Key Laboratory, Tibetan Plateau Juema Research Centre, Xining, 810007 China

**Keywords:** Microbial communities, Soil microbiology

## Abstract

Plant roots and soil prokaryotes primarily interact with each other in the rhizosphere. Changes in the rhizosphere prokaryotic structure are influenced by several factors. In this study, the community structure of the *Potentilla anserina* L. rhizosphere prokaryotes was identified and evaluated by high-throughput sequencing technology in different continuous cropping fields and developmental stages of the plant. In total, 2 archaeal (Euryarchaeota and Thaumarchaeota) and 26 bacterial phyla were identified in the *P. anserina* rhizosphere. The bacterial community was mainly composed of Acidobacteria, Actinobacteria, Bacteroidetes, Chloroflexi, Gemmatimonadetes, Planctomycetes, Proteobacteria, and Verrucomicrobia. Moreover, the prokaryotic community structure of the rhizosphere varied significantly during plant development. Our results provide new insights into the dynamics of the *P. anserina* rhizosphere prokaryotic community and may provide useful information for enhancing the growth and development of *P. anserina* through artificial control of the soil prokaryotes.

## Introduction

*Potentilla anserina* L. (*Argentina anserina*), affiliated with Rosaceae *Potentilla*, is a typical stoloniferous and rosulate clonal plant. It is widely distributed in China, particularly in extremely cold or high altitude areas, such as Qinghai, Tibet, Sichuan, and Gansu^1^. It has been consumed as a highly valued tonic food and folk medicine^2^. Its tuberous roots have been applied in herbal medicine due to their potential to promote body fluid production, thereby relieving thirst, strengthening the spleen and stomach, and invigorating the blood, among other beneficial health effects^3^. In addition, modern pharmacological studies have revealed that the tuberous roots of *P. anserina* have multiple properties, including antioxidant, anti-aging, anti-inflammatory, antihyperlipidemic, hepatoprotective, and immunomodulatory effects^4–9^.

Soil microorganisms are an important component of terrestrial ecosystems, and agricultural productivity in particular is closely related to the activities of soil microorganisms^10^. Plant growth, development, and overall health are affected by the activities of their associated microbes^11–13^, resulting in either enhanced or compromised performance^14^. Studies have demonstrated that members of the rhizosphere microbiome hold broad beneficial properties that contribute to preventing soil-borne diseases^15^; obtaining nutrients; promoting the availability of mineral fertilizers, such as nitrogen and phosphorus; improving stress resistance; regulating stress hormones; and promoting detoxification^10,16–20^.

Rhizosphere microbial diversity is also determined by the genotype of the host plant^21–28^ and soil physicochemical characteristics, including nutrient composition (nitrogen and phosphorus contents), pH value, the ratio of carbon to nitrogen, and texture^26,29–33^. Evidence suggests that novel plant varieties capable of producing new carbon compounds rapidly select and accumulate bacteria capable of metabolizing these compounds during rhizosphere development^34^. Moreover, rhizosphere microbial communities appear to vary with changes in plant developmental stages^35–38^. Indeed, some exudates secreted by the roots of various plants can affect the structure of the rhizosphere microbiome and are known to act as substrates, attractants, stimulants, inhibitors, repellents, or signaling molecules^39–47^. Therefore, resident plants have the ability to autonomously select rhizosphere biodiversity and can shape and reorganize the rhizosphere microbial community^48^.

At present, there is a concerted understanding of the dynamic interactions between plants and soil microorganisms that are important in agricultural systems. However, our knowledge of the relationship between plant development and rhizosphere prokaryotic community structure is limited. Here, 36 *P. anserina* rhizosphere soil samples were collected at 4 distinct stages of plant development (flowering, vegetative, harvest-enlargement stage of the tuberous root, and germinating) in 3 agricultural field environments. Using high-throughput sequencing of the *16S* ribosomal RNA (rRNA) gene, we aimed to characterize the structure of the rhizosphere prokaryotic community associated with *P. anserina*.

## Results

### Rhizosphere prokaryotic community structure associated with *P. anserina*

Next-generation sequencing analysis of the rhizosphere prokaryotic community achieved 2,733,491 high-quality *16S* rRNA gene sequence reads. After an effort equalize sampling, 2,449,624 reads were retained for further analysis with an average of 68,045 reads per sample (min = 32,909; max = 90,611). These reads clustered into 2809 operational taxonomic units (OTUs) at a 3% dissimilarity threshold.

**Figure 1:**
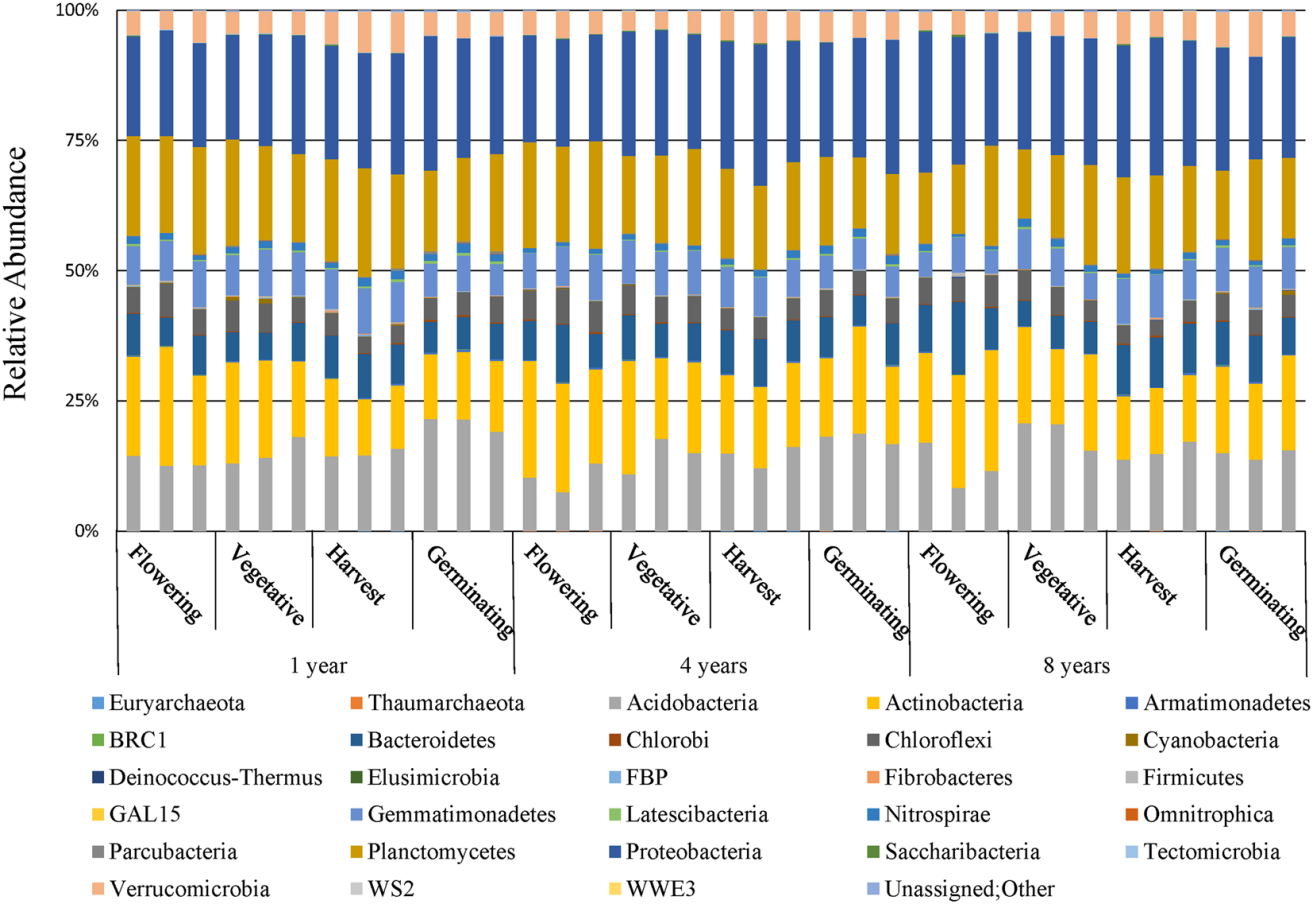
Relative abundance (%) of the major bacterial and archaea phyla present in the rhizosphere prokaryotic community at each developmental stage of *Potentilla anserina* L.

**Figure 2:**
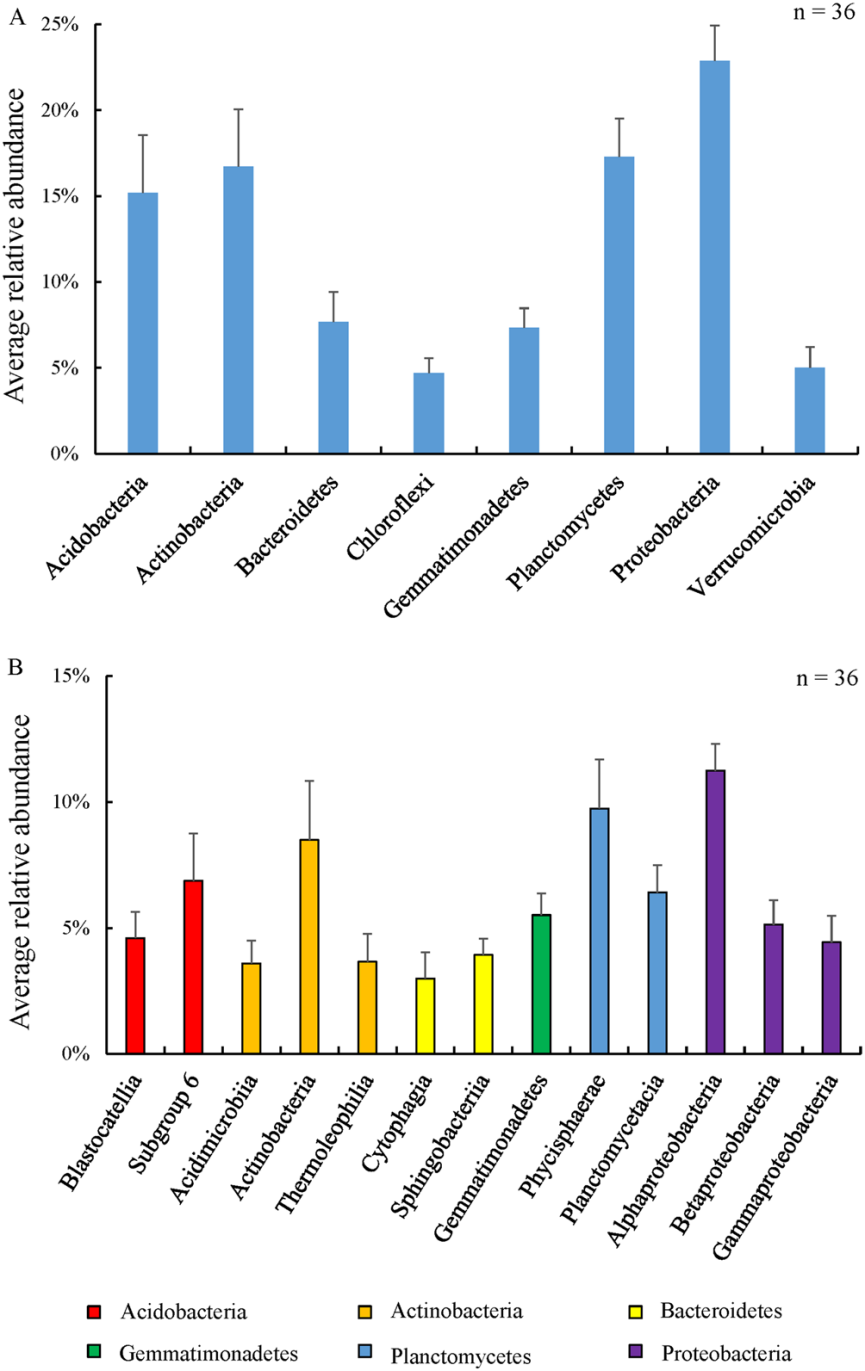
Average relative abundance of prokaryotic taxa in samples collected from rhizosphere soil at each developmental stage of *Potentilla anserina*. Average relative abundance was estimated for each prokaryotic taxon by dividing the total relative abundance across all samples by the number of samples (n = 36). The error bars show the standard deviation (calculation across 36 samples) for each average value. (**A**) Prokaryotic phyla with average relative abundance > 1% and (**B**) prokaryotic classes with average relative abundance > 1%.

Alignment of the identified OTUs with bacteria and archaea rRNA data from the SILVA database revealed that the soil prokaryotic community comprised 28 phyla (unclassified phylotypes were not included in the analysis), among which two (Euryarchaeota and Thaumarchaeota) belonged to archaea domain (Fig. 1). The abundance of each phylum varied in *P. anserina* rhizosphere soil samples, but Acidobacteria, Actinobacteria, Bacteroidetes, Chloroflexi, Gemmatimonadetes, Planctomycetes, Proteobacteria, and Verrucomicrobia were the 8 dominant phyla (relative abundance ≥ 1) within the 4 different developmental stages and 3 different fields, accounting for 96.75% of all prokaryotic taxa (Fig. 2A). Proteobacteria were the most abundant and accounted for 22.87% of the total 685,587 OTUs followed by Planctomycetes, Actinobacteria, Acidobacteria, Bacteroidetes, Gemmatimonadetes, Verrucomicrobia, and Chloroflexi, which represented 17.28%, 16.73%, 15.19%, 7.66%, 7.33%, 5.00%, and 4.69% of the OTUs, respectively. The OTUs within these 26 bacterial phyla were further classified into 86 classes, 131 orders, 223 families, and 353 genera.

Within the eight dominant phyla, several taxa were maintained during the entire sampling period regardless of the location, including Alphaproteobacteria, Betaproteobacteria, and Gammaproteobacteria from the Proteobacteria phylum as well as Phycisphaerae and Planctomycetacia from the Planctomycetes phylum (Fig. 2B).

### Rhizosphere prokaryotic β-diversity associated with *P. anserina*

**Figure 3:**
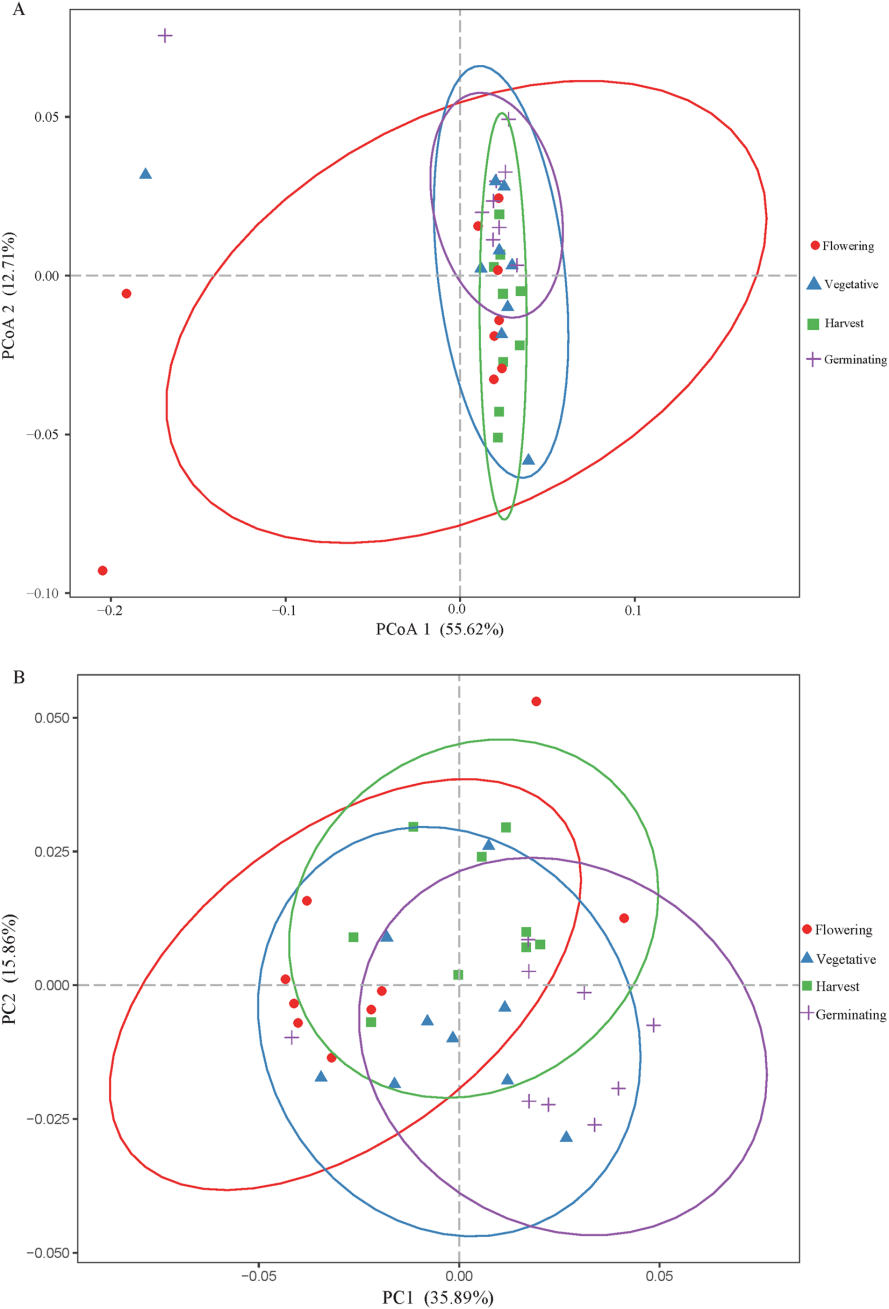
Factors influencing rhizosphere prokaryotic β-diversity. Variation in unweighted UniFrac dispersion (**A**) and weighted UniFrac dispersion (**B**) based on each developmental stage of *Potentilla anserina* (*p* < 0.05).

For the analysis of multivariate homogeneity among groups, the analysis of similarities (ANOSIM) test was performed, and the results showed that there were significant differences between the developmental stages (*p* = 0.001). Unweighted (Fig. 3A) and weighted (Fig. 3B) UniFrac distance metrics were used to estimate the rhizosphere prokaryotic β-diversity and identify dissimilarities between the different developmental stages. The first two principle components explained 68.33% (principle coordinate analysis PCoA 1 + PCoA 2) and 51.75% (PCA 1 + PCA 2) of the data variability, respectively. These results clearly demonstrate that the rhizosphere prokaryotic community had different structures throughout various plant developmental stages. Further analyses revealed significant differences in six phyla—GAL15, Latescibacteria, Nitrospirae, Omnitrophica, Planctomycetes, and WWE3—from rhizosphere samples collected from different continuous cropping years fields, whereas all of the other phyla did not change significantly (Supplementary Fig. S1). Except for WWE3, the other five phyla showed the highest abundances in the rhizosphere soil of continuous cropping for 1 year while Latescibacteria, Nitrospirae, and Planctomycetes had the lowest abundances in the rhizosphere soil of continuous cropping for 8 years (Supplementary Fig. S1). These results reveal that while the soil prokaryotic community as a whole was maintained, soil-specific prokaryotic phyla were influenced by continuous cropping years. Moreover, significant differences in prokaryotic community composition were also observed between the different plant developmental stages, affecting 12 prokaryotic phyla (in particular: Euryarchaeota, Acidobacteria, Actinobacteria, Armatimonadetes, BRC1, Bacteroidetes, Chloroflexi, Fibrobacteres, Latescibacteria, Parcubacteria, Saccharibacteria, and Verrucomicrobia) (Supplementary Fig. S2), while all other phyla did not change significantly. These data indicate that the rhizosphere prokaryotic community was influenced by plant development.

**Figure 4:**
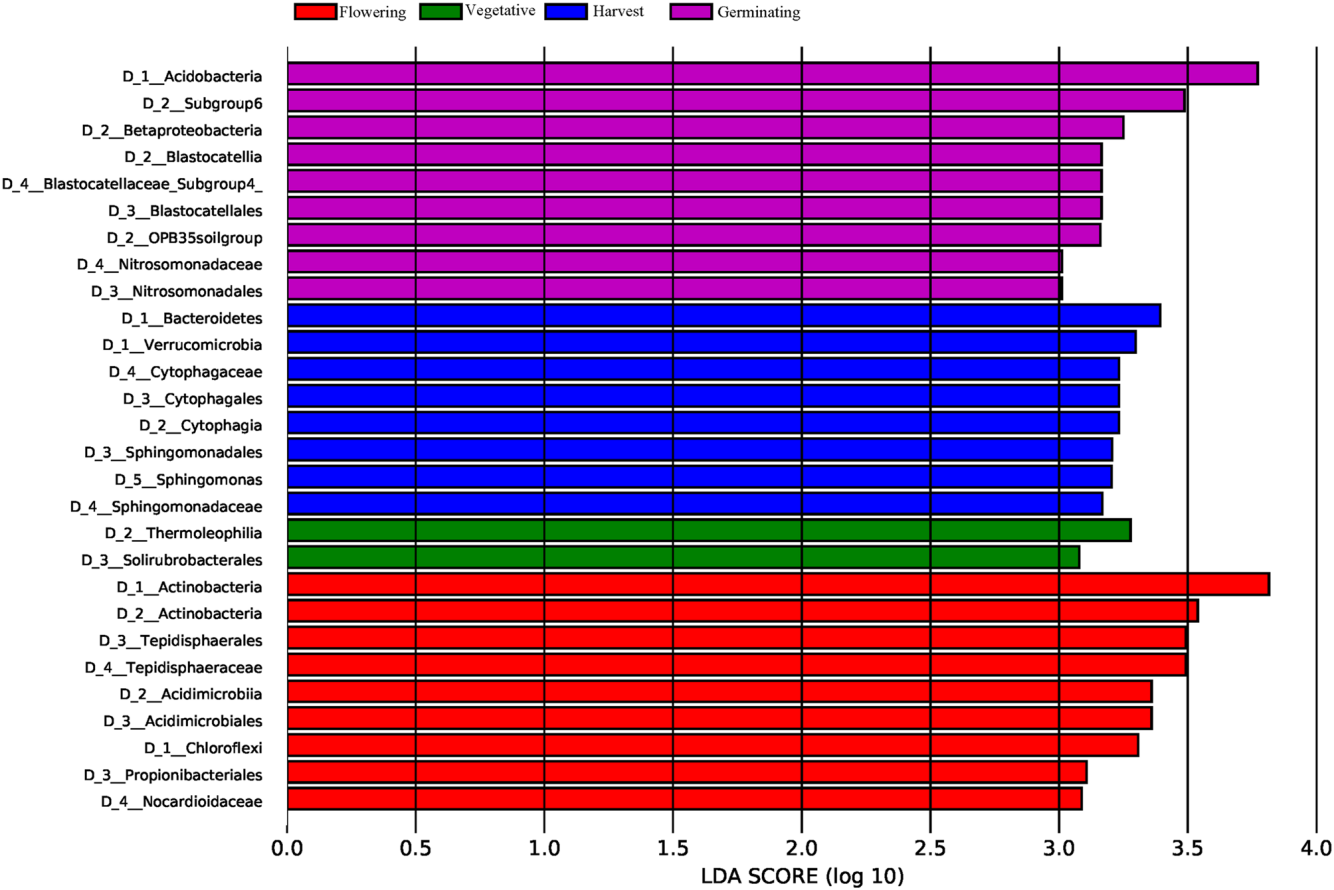
Linear discriminant analysis effect size (LEfSe) identifying the most differentially abundant taxa among the various developmental stages of *Potentilla anserina*. Only the taxa identified as meeting a significant LDA threshold of > 3 are shown.

Next, linear discriminant analysis effect size (LEfSe) analysis was performed to identify the taxonomical lineages that were significantly influenced by the plant developmental process (Fig. 4). The data showed that rhizosphere samples from flowering plants harbored more prokaryotes of the phyla Actinobacteria and Chloroflexi; classes Actinobacteria and Acidimicrobiia; orders Tepidisphaerales, Acidimicrobiales, and Propionibacteriales; and families Tepidisphaeraceae and Nocardiodaceae compared to the rhizosphere associated with other plant stages. The harvest stage was characterized by the presence of more prokaryotes of the phyla Bacteroidetes and Verrucomicrobia; class Cytophagia; orders Cytophagales and Sphingomonadales; families Cytophagaceae and Sphingomonadaceae; and genus *Sphingomonas* while the phylum Acidobacteria; classes Subgroup 6, Betaproteobacteria, Blastocatellia, and OPB35soilgroup; orders Blastocatellales and Nitrosomonadales; and families Blastocatellaceae_Subgroup4 and Nitrosomonadaceae were more abundant in the germinating stage. Only the class Thermoleophilia and order Solirubrobacterales of the rhizosphere samples associated with plants in the vegetative stage differed significantly from samples associated with other plant stages (Fig. 4). These results suggest that *P. anserina* can select the prokaryotes that populate the soil at different stages of its development, presumably for attaining specific benefits.

### Rhizosphere prokaryotic α-diversity associated with *P. anserina*

**Figure 5:**
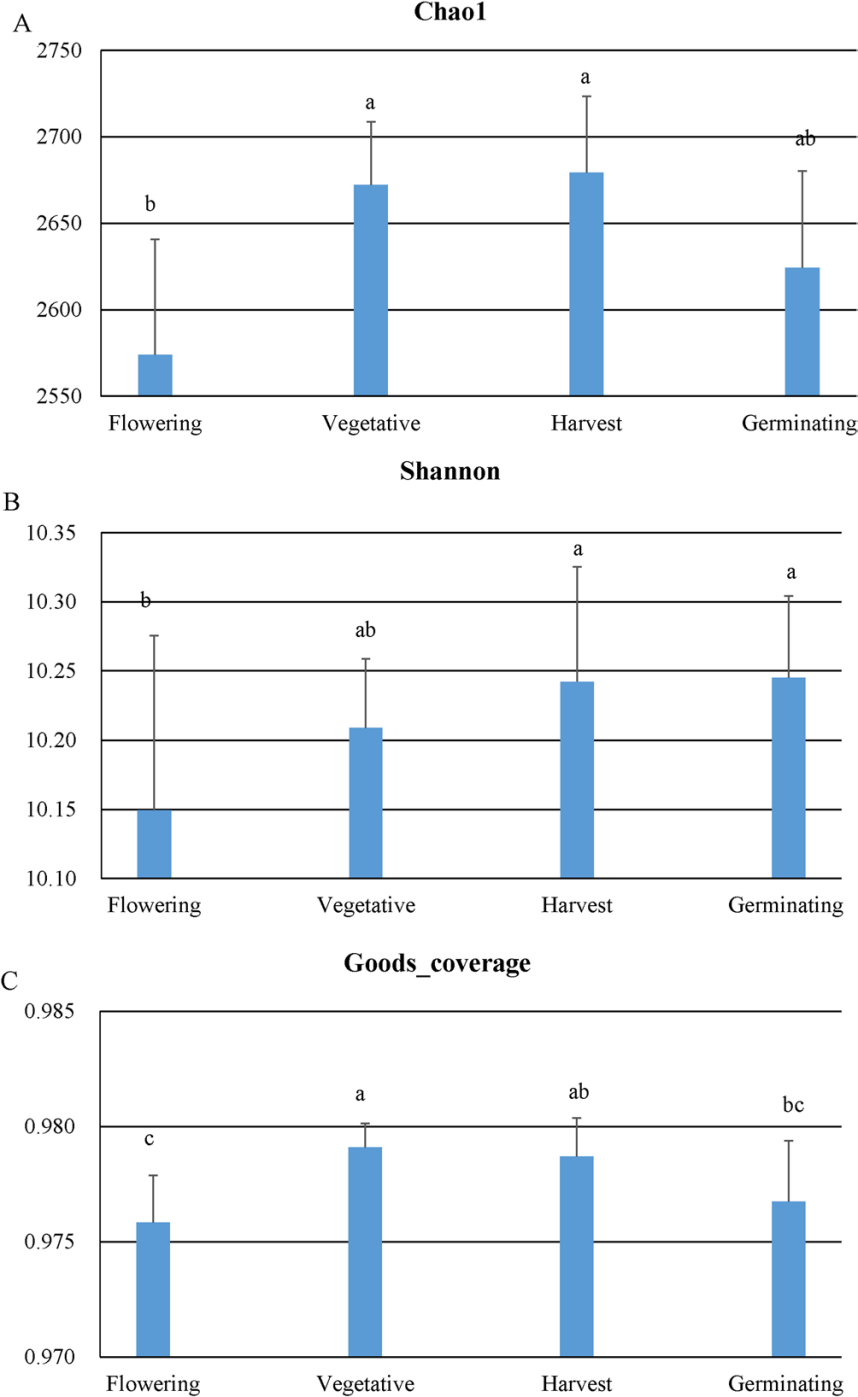
Prokaryotic diversity that significantly changes with *Potentilla anserina* development. The bars with different letters are significantly different (analysis of variance Tukey post-hoc *p* < 0.05) from one another. Graphs show mean ± standard error. (**A**) Chao1 index, (**B**) Shannon index and (**C**) Goods_coverage.

The α-diversity analysis revealed that the prokaryotic community richness and diversity varied widely among the samples (Supplementary Table S1). The Good’s coverage values were > 0.95 in all samples, indicating that the sequencing depth was sufficient to investigate the various rhizosphere prokaryotic communities. We observed substantial variation in the prokaryotic diversity of taxa between different developmental stages (Fig. 5). The vegetative and harvest stages had the largest community richness (Chao1) and Good’s coverage compared to the other stages, whereas the flowering stage had the lowest (*p* < 0.05). Although no statistically significant differences with respect to overall community characteristics were seen between the three different continuous cropping field soil samples, the prokaryotic community in continuous cropping for one year had the largest community diversity (Shannon) compared to the other continuous cropping years during the vegetative stage (*p* < 0.05). These findings suggest that both the overall structure of the rhizosphere prokaryotic community and specific prokaryotes changed throughout the plant developmental stages.

### Impact of soil environmental factors on the rhizosphere prokaryotes

Spearman’s rank correlation test was performed in order to clarify the relationship between environmental factors and prokaryotic diversity (Table 1). For the rhizosphere prokaryotic communities, the Chao1 index was negatively correlated with the available potassium (AK) content and temperature, but positively correlated with the accumulated precipitation (P) (*p* < 0.05). Similarly, the Shannon index was also negatively correlated with temperature (*p* < 0.05).

**Table 1:** Relationship of rhizosphere prokaryotic diversity with the measured environmental variables.

Diversity index		TN	TK	TP	AN	AK	AP	M	T	P
Observed_species	*r*	0.078	− 0.286	− 0.184	− 0.288	− 0.264	− 0.297	− 0.172	− 0.386*	0.402*
Chao1	*r*	0.141	− 0.290	− 0.237	− 0.282	− 0.384*	− 0.321	− 0.202	− 0.407*	0.430**
Shannon	*r*	0.166	− 0.145	0.123	0.039	− 0.005	− 0.015	− 0.326	− 0.414*	− 0.093
Simpson	*r*	0.258	− 0.008	0.247	0.241	0.035	0.073	− 0.249	− 0.323	− 0.263
Goods_coverage	*r*	− 0.039	− 0.307	− 0.251	− 0.287	− 0.154	− 0.331*	− 0.107	− 0.323	0.478**

The Spearman’s rank correlation coefficient (*r*) are shown. Correlations where *p* < 0.05 were considered to be significant.

*TN* total nitrogen, *TK* total potassium, *TP* total phosphorus, *AN* available nitrogen, *AK* available potassium, *AP* available phosphorus, *M* moisture, *T* temperature, *P* accumulated precipitation 30 days before sampling time.

*p < 0.05 and **p < 0.01.

Next, the relationships between prokaryotic composition and environmental factors were evaluated with a focus on taxa with a relative abundance at the phylum level (Table 2) and the top 15 at the genus level (Supplementary Table S2). The Monte Carlo permutations results at the OTU level showed that the total nitrogen (TN), total phosphorous (TP), and available nitrogen (AN) soil contents had a highly significant influence on rhizosphere prokaryotic communities (*p* < 0.001), whereas the total potassium (TK) and available phosphorus (AP) contents had a significant influence (*p* < 0.05) (Table 3). These findings suggest that precipitation, temperature, soil water and nitrogen, phosphorus, potassium content represent important contributing factors for regulation of the rhizosphere prokaryotes.

**Table 2:** Correlations between the measured environmental variables and relative abundances of rhizosphere prokaryotic phyla.

Classification		TN	TK	TP	AN	AK	AP	M	T	P
Euryarchaeota	*r*	− 0.041	− 0.271	− 0.137	− 0.115	− 0.103	− 0.243	− 0.317	− 0.624**	− 0.055
Thaumarchaeota	*r*	− 0.274	0.177	− 0.091	0.134	0.360*	0.268	0.070	0.326	− 0.340*
Acidobacteria	*r*	0.077	− 0.084	− 0.126	0.018	0.033	− 0.212	− 0.545**	− 0.347*	− 0.084
Actinobacteria	*r*	− 0.327	0.196	0.176	0.001	0.280	0.486**	0.544**	0.732**	0.019
Armatimonadetes	*r*	− 0.035	− 0.365*	− 0.195	− 0.084	0.169	0.044	− 0.518**	− 0.368*	− 0.244
BRC1	*r*	0.042	− 0.028	− 0.094	− 0.005	− 0.227	− 0.146	0.450**	0.125	0.146
Bacteroidetes	*r*	− 0.112	0.133	− 0.171	− 0.109	− 0.127	− 0.159	0.003	− 0.242	− 0.127
Chlorobi	*r*	0.025	− 0.168	− 0.071	0.118	− 0.119	0.012	− 0.044	− 0.005	− 0.349*
Chloroflexi	*r*	− 0.227	0.288	0.157	0.160	0.344*	0.550**	0.478**	0.770**	− 0.022
Cyanobacteria	*r*	0.064	− 0.140	0.037	0.079	− 0.152	0.149	0.236	0.093	0.196
Deinococcus-Thermus	*r*	0.008	− 0.070	− 0.078	− 0.091	− 0.318	− 0.144	0.166	0.012	0.147
Elusimicrobia	*r*	0.108	− 0.123	0.105	− 0.052	0.089	0.147	− 0.217	− 0.108	0.058
FBP	*r*	− 0.090	0.372*	0.065	− 0.048	0.084	− 0.112	0.197	0.065	0.098
Fibrobacteres	*r*	0.230	0.025	0.215	0.300	− 0.108	− 0.064	− 0.125	− 0.402*	− 0.285
Firmicutes	*r*	0.116	0.138	0.509**	0.283	0.179	0.442**	0.153	0.146	− 0.172
GAL15	*r*	0.192	0.239	0.144	0.191	0.064	− 0.101	− 0.121	− 0.107	− 0.011
Gemmatimonadetes	*r*	0.046	− 0.169	− 0.053	− 0.265	− 0.319	− 0.140	0.126	− 0.091	0.373*
Latescibacteria	*r*	0.388*	− 0.242	0.172	0.213	0.102	0.051	− 0.528**	− 0.371*	− 0.239
Nitrospirae	*r*	0.303	− 0.013	0.215	0.252	0.259	0.069	− 0.309	− 0.232	− 0.062
Omnitrophica	*r*	0.448**	0.049	0.402*	0.376*	0.129	0.127	− 0.174	− 0.089	− 0.194
Parcubacteria	*r*	0.396*	− 0.289	− 0.148	− 0.005	− 0.267	− 0.428**	− 0.491**	− 0.646**	0.191
Planctomycetes	*r*	0.327	0.115	0.429**	0.271	0.282	0.216	0.371*	0.143	0.096
Proteobacteria	*r*	− 0.175	− 0.299	− 0.490**	− 0.380*	− 0.468**	− 0.492**	− 0.395*	− 0.464**	0.055
Saccharibacteria	*r*	− 0.149	0.090	− 0.090	− 0.195	− 0.216	− 0.216	0.412*	− 0.031	0.373*
Tectomicrobia	*r*	0.076	0.317	0.366*	0.389*	0.153	0.283	0.247	0.323	− 0.096
Verrucomicrobia	*r*	0.190	− 0.179	− 0.099	0.041	− 0.112	− 0.252	− 0.462**	− 0.672**	− 0.306
WS2	*r*	0.357*	0.083	− 0.017	0.307	− 0.027	− 0.138	− 0.288	− 0.246	− 0.213
WWE3	*r*	− 0.499**	− 0.120	− 0.500**	− 0.589**	− 0.215	− 0.331*	0.058	− 0.032	0.359*

The Spearman’s rank correlation coefficient (*r*) are shown. Correlations where *p* < 0.05 were considered to be significant.

*TN* total nitrogen, *TK* total potassium, *TP* total phosphorus, *AN* available nitrogen, *AK* available potassium, *AP* available phosphorus, *M* moisture, *T* temperature, *P* accumulated precipitation 30 days before sampling time.

*p < 0.05 and **p < 0.01.

**Table 3:** Correlations between environmental variables and rhizosphere prokaryotic composition (OTU level) assessed by the Monte Carlo permutation test (999 permutations) for canonical correspondence analysis.

Variables	RDA1	RDA2	*r*^*2*^	*p*
TN	0.18879	− 0.98202	0.3917	0.001***
TK	− 0.70846	− 0.70575	0.1755	0.036*
TP	− 0.47546	− 0.87974	0.4319	0.001***
AN	− 0.65334	− 0.75706	0.3718	0.001***
AK	− 0.31669	− 0.94853	0.0602	0.335
AP	− 0.86517	− 0.50148	0.2364	0.016*

*p < 0.05, **p < 0.01, ***p < 0.001.

## Discussion

### Structure and potential function of the *P. anserina* rhizosphere prokaryotic community

A more detailed look at the assembled rhizosphere prokaryotic communities throughout plant development revealed that a core prokaryotic/bacterial microbiome was established, which comprised Actinobacteria, Bacteroidetes, Chloroflexi, Gemmatimonadetes, Planctomycetes, Proteobacteria, and Verrucomicrobia as previously observed in *Arabidopsis*^35,49,50^. In addition, the present study demonstrated that Acidobacteria were also consistently present throughout plant development (Fig. 2). Proteobacteria were the most abundant phylum within the rhizosphere of *P. anserina*. Some strains of Proteobacteria can promote plant growth by symbiotically fixing nitrogen^51,52^, such as *Sphingomonas* (its relative abundance was the second highest in the present study) and *Dokdonella*, which are very important genera for nitrogen and carbon cycling^53–55^. Studies have shown that Actinobacteria are involved in the soil phosphorous cycle^56,57^. Moreover, a previous study reported that bacteria, such as Proteobacteria and Actinobacteria, prefer nutrient-rich environments where they can grow rapidly^58,59^. Among the prokaryotes of the *P. anserina* soil rhizosphere, the abundances of Proteobacteria and Actinobacteria were first and third, respectively, which is in accordance with previous studies. Acidobacteria was the fourth most abundant taxon in the studied soils, and Acidobacteria *Subgroup_6* was the most abundant genus in this phylum (fifth out of all prokaryotic genera), which may be a response to nitrogen availability^60^. Studies have indicated that the bacteria of the phylum Chloroflexi can participate in the carbon and nitrogen cycle via respiration of sugars, fermentation, carbon dioxide fixation, and nitrite oxidation^61–63^. Some *Pseudomonas* and *Bacillus* were also identified within the rhizosphere of *P. anserina*, and previous studies have shown that these genera can promote plant growth through nutrient acquisition, reducing abiotic or biotic stress, and phytohormone production^16,64^. Ultimately, the presence of so many prokaryotic taxa in the rhizosphere, most of which are unculturable, prevented us from understanding the role of individual prokaryotes in *P. anserina* growth^65–67^.

### Plant developmental changes affect the rhizosphere prokaryotic community

Bray–Curtis community dissimilarity analysis of the overall rhizosphere prokaryotic community throughout *P. anserina* development revealed that the prokaryotic community was significantly different at various developmental stages (Fig. 3). These results are in agreement with previous reports as the rhizosphere microbiome communities change according to a plant developmental gradient^35–38,68^. For example, Baudoin’s^36^ results argue in favor of a greater influence of the maize rhizosphere environment on bacterial metabolic potentialities, which were primarily based on the developmental state of the plant. In addition, the α-diversity of the prokaryotic community significantly changed with respect to the developmental stages (Fig. 5), and the prokaryotic community at the flowering stage was significantly different from the other developmental stages (Chao1, *p* = 0.001). Previous reports also showed that major modifications were recorded at the first reproductive stage (flowering) of *Medicago truncatula* for both bacterial and fungal communities^38^. For instance, during the flowering stage, genes involved in the synthesis of streptomycin were significantly induced^35^ and a strengthening of defensive proteins secreted by the root system took place^69^, which effectively inhibited bacteria. On the contrary, during the vegetative stage, the significantly stronger rhizosphere effect toward bacteria over fungi could be ascribed to the expected higher release of rhizodeposits, primarily as soluble root exudates, which are more favorable to bacteria^38^. Studies on *Arabidopsis thaliana* have shown that the microbial community structure differs the most at the seedling stage^35^, which is inconsistent with our results, possibly because different species of plants secrete very different root exudates at various growth stages^29^.

Phyla, such as Acidobacteria, Actinobacteria, Bacteroidetes, Chloroflexi, and Verrucomicrobia (Supplementary Fig. S2), and specific genera followed distinct patterns associated with plant development. The community dissimilarity analysis revealed that the structures of the prokaryotic communities changed significantly among the different plant developmental stages (Fig. 3); this was particularly noticeable for Actinobacteria, Acidobacteria, and Bacteroidetes (Fig. 4), which is in agreement with previous reports^35,49,50^. Additionally, previous research on the rhizosphere microbiome revealed that unique transcripts were significantly expressed at different stages of plant development^35^. Altogether, the plant secretes specific phytochemicals in the roots at distinct stages of development, thereby coordinating the structure of the rhizosphere microbial community and achieving specific results^35,37,38,49^.

### Soil environmental factors influence rhizosphere prokaryotic composition

Environmental conditions have been shown to significantly impact the microbial communities that populate the soil^70,71^. To fully investigate the impact of the environment on the rhizosphere prokaryotes of *P. anserina*, it is necessary to identify the key environmental factors that may be involved. The present study demonstrated that soil environmental factors were significantly correlated with the rhizosphere prokaryotic community structure associated with *P. anserina* (Tables 1, 2 and 3 and Table S2), which was in line with previous studies reporting on the significant roles of nitrogen and phosphorus in modulating the soil microbiome^26,70–73^. Among the eight identified dominant phyla, Acidobacteria, Actinobacteria, Chloroflexi, Gemmatimonadetes, Planctomycetes, Proteobacteria, and Verrucomicrobia were significantly affected by nitrogen, phosphorus, potassium, moisture, temperature and accumulated precipitation (Table 2). Furthermore, 10 of the 15 genera with the highest relative abundances were significantly associated with multiple environmental factors (Supplementary Table S2). In summary, soil environmental factors have a significant influence on the structure of the rhizosphere prokaryotic community and a selective effect on rhizosphere prokaryotes, which is in agreement with previously reported data^26^. Interestingly, Bacteroidetes were not sensitive to environmental factors, but their relative abundance varied significantly at different plant growth stages (Supplementary Fig. S2). It is possible that the different root exudates throughout the four developmental stages can promote the conversion of microbial groups^44–46^.

Interaction between plants and microbes plays an important role in agricultural systems^11^. With this in mind, our future investigations will focus on the functions of rhizosphere prokaryotes. In particular, we aim to improve our understanding of the beneficial and harmful impacts of specific plant prokaryotic communities in order to pave the way for improved agricultural production. Such findings will enhance our understanding of these interactions and, in the future, provide evidence for the sustainable use of farmland to meet the needs of more efficient and productive agriculture by selectively enhancing the development of prokaryotic strains with beneficial functions.

## Materials and methods

### Sample collection

This study was performed in Huangyuan County, Qinghai Province, China. Crops were planted using *P. anserina* seeds in the Sitan village, Tuergan village, and Kesuer village. No plant protection methods were used, enabling the study of natural populations, and the fields were weeded by hand and irrigated as necessary. The sowing information is presented in Table 4. Rhizosphere soil samples of *P. anserina* were collected at four different growth stages: flowering (June 17, 2018), vegetative (root tuber) (September 8, 2018), harvest (root tuber) (November 10, 2018), and germinating (April 29, 2019). The sampling weather information is shown in Supplementary Table S3.

**Table 4:** Sowing information of *Potentilla anserina* in Huangyuan County, Qinghai Province, China.

Location	Coordinates	Altitude (m)	Planting areas (acres)	Sowing time
Sitan village (S)	36°31′31″N 101°7′51″E	3104	16.968	May 2017
Tuergan village (T)	36°31′30″N 101°8′55″E	3031	3.295	May 2014
Kesuer village (K)	36°32′40″N 101°10′24″E	2939	1.812	May 2010

In the present study, the classical definition of the rhizosphere described by Chaparro et al.^35^ was used. Soil samples were collected from the surface layer (0–30 cm) of a field attached to the roots. We sampled three quadrats (3 × 3 m) in each field, and the distance between each quadrat was > 10 m. From each quadrat, the rhizosphere soil of five plants with the same growth potential was collected, and the five sub-samples were mixed to create one sample. The sampled plants were labeled for identification at each sampling time. Three biological replicates were selected from each of the 3 sample quadrats in each field across the 4 developmental stages (36 samples). The samples were air dried, cleaned of plant debris, thoroughly homogenized, and stored at − 80 ℃ for future use.

### Soil chemical analysis

Measurements of AP, TP, AN, TN, AK, and TK were performed at the Analytical Testing Center of the Northwest Institute of Plateau Biology, Chinese Academy of Sciences. The results are summarized in Supplementary Table S4.

### DNA extraction and library generation

DNA was extracted from the soil samples (0.5 g) using a QIAamp PowerFecal DNA Kit (Qiagen, Hilden, Germany) according to the manufacturer’s instructions. Subsequently, the primers 460F (5′-CCTACGGGNBGCASCAG-3′) and 460R (5′-GACTACNVGGGTATCTAATCC-3′) were used to amplify the V3–V4 hyper-variable region of the *16S* rRNA gene of bacteria and archaea^74^. PCR reactions were performed in a 25 μL mixture containing 5 μL of (5×) GC Buffer, 0.5 μL of KAPA dNTP Mix, 0.5 μL of KAPA HiFi HotStart DNA Polymerase (Kapa Biosystems, Wilmington, MA, USA), 0.5 μL of each primer (10 pM), and a 50–100 ng of sample DNA. The PCR reaction cycling protocol was as follows: 95 °C for 3 min, followed by 25 cycles at 95 °C for 30 s, 55 °C for 30 s, 72 °C for 30 s, and a final extension at 72 °C for 5 min. PCR clean up with AMPure XP beads (Beckman Coulter, Brea, CA, USA) was performed to purify the *16S* V3–V4 amplicon from free primers and primer dimer species^75^. The purified product underwent a new PCR amplification using the same primer as before, which had been attached an eight-base sequence unique to each sample. The PCR reactions were performed in a 25 μL mixture containing 5 μL of (5×) GC Buffer, 0.75 μL of KAPA dNTP Mix, 0.5 μL of KAPA HiFi HotStart DNA Polymerase, 1.5 μL of each primer (10 pM), and 5 μL of the purified product. The PCR reaction cycling protocol was as follows: 95 °C for 3 min followed by 8 cycles at 95 °C for 30 s, 55 °C for 30 s, 72 °C for 30 s, and a final extension at 72 °C for 5 min^75^. The amplicons were subsequently purified using AMPure XP beads to clean up the final library before quantification. Lastly, the purified amplicons were pooled in equimolar concentrations and paired-end sequenced (2 × 250) on an Illumina HiSeq platform (Illumina, San Diego, CA, USA) according to the standard protocols. The raw reads were stored in the National Center for Biotechnology Information Sequence Read Archive database.

### Bioinformatics analysis

The Fast Length Adjustment of SHort reads software tool was used to merge paired-end reads from the next-generation sequencing analysis^76^. Low quality reads were filtered using the fastq_quality_filter algorithm (-p 90 -q 25 -Q33) of the FASTX Toolkit (v0.0.14, http://hannonlab.cshl.edu/fastx_toolkit/index.html), and chimera reads were removed by USEARCH (64 bit, v8.0.1517, https://www.drive5.com/usearch/). The number of reads for each sample was normalized based on the smallest size sample by random subtraction. OTUs were aligned using the UCLUST algorithm with 97% identity and taxonomically classified using the SILVA *16S* rRNA database v128^77^ (https://www.arb-silva.de/documentation/release-128/).

### Statistical analysis

We used custom R scripts in R software (v2.13.2) to calculate the percentage of classifiable reads. Differences in prokaryotic community composition within the sampled locations were analyzed by one-way analysis of variance and Tukey's post hoc test, and correlations between prokaryotic diversity, prokaryotic community structure, and environmental variables were determined using Spearman’s rank correlation test. These statistical analyses were performed using IBM SPSS Statistics 20 software (IBM SPSS, Armonk, NY, USA). The α- and β-diversities were generated via the Quantitative Insights Into Microbial Ecology (http://qiime.org) pipeline and calculated based on the Bray–Curtis index^78^. Centroids of distance matrices were tested using ANOSIM to assess the multivariate homogeneity of groups. We used the LEfSe method to identify species that showed statistically significant differential abundances between groups^79^. To further investigate the effects of environmental factors on the prokaryotes and identify the key factors, a significance analysis was performed using Monte Carlo permutations. A *p* value < 0.05 was considered to be statistically significant.

## Supplementary Information

Supplementary Information
