# Supplementary material for: Potentilla anserina L. developmental changes affect the rhizosphere prokaryotic community
Source: Sci Rep. 2021 Feb 2;11:2838. doi: 10.1038/s41598-021-82610-9 (PMC7854623; doi:10.1038/s41598-021-82610-9)
Supplement: Supplementary file 1 — Supplementary Information [file 41598_2021_82610_MOESM1_ESM.docx]

**Supplementary Information:**

*Potentilla anserina* L. developmental changes affect the rhizosphere prokaryotic community

Yaqiong Wang^1,2,3*^, Yuxi Liu^1^, Xue Li^1^, Xiaoyan Han^1^, Zhen Zhang^1^, Xiaoling Ma^1^, Junqiao Li^1,2,3*^

^1^School of Ecology, Environment and Resources, Qinghai Nationalities University, Xining, 810007, China

^2^Qinghai Provincial Key Laboratory of High-value Utilization of Characteristic Economic Plants, Xining, 810007, China

^3^Qinghai Provincial Biotechnology and Analytical Test Key Laboratory, Tibetan Plateau Juema Research Centre, Xining, 810007, China

*Correspondence to: Yaqiong Wang and Junqiao Li

Qinghai Nationalities University, Bayi Road, Xining, Qinghai 810007, China.

Telephone: 86-18297178818

Email: [wangyaqiong727@163.com](mailto:wangyaqiong727@163.com) and 18297178818@163.com

**Supplementary Figure S1.** Prokaryotic phyla that significantly change with different continuous cropping years of *Potentilla anserina*. (**A**) GAL15, (**B**) Latescibacteria, (**C**) Nitrospirae, (**D**) Omnitrophica, (**E**) Planctomycetes, and (**F**) WWE3. The bars with different letters are significantly different (analysis of variance Tukey post-hoc p < 0.05) from one another. Graphs show mean ± standard error.


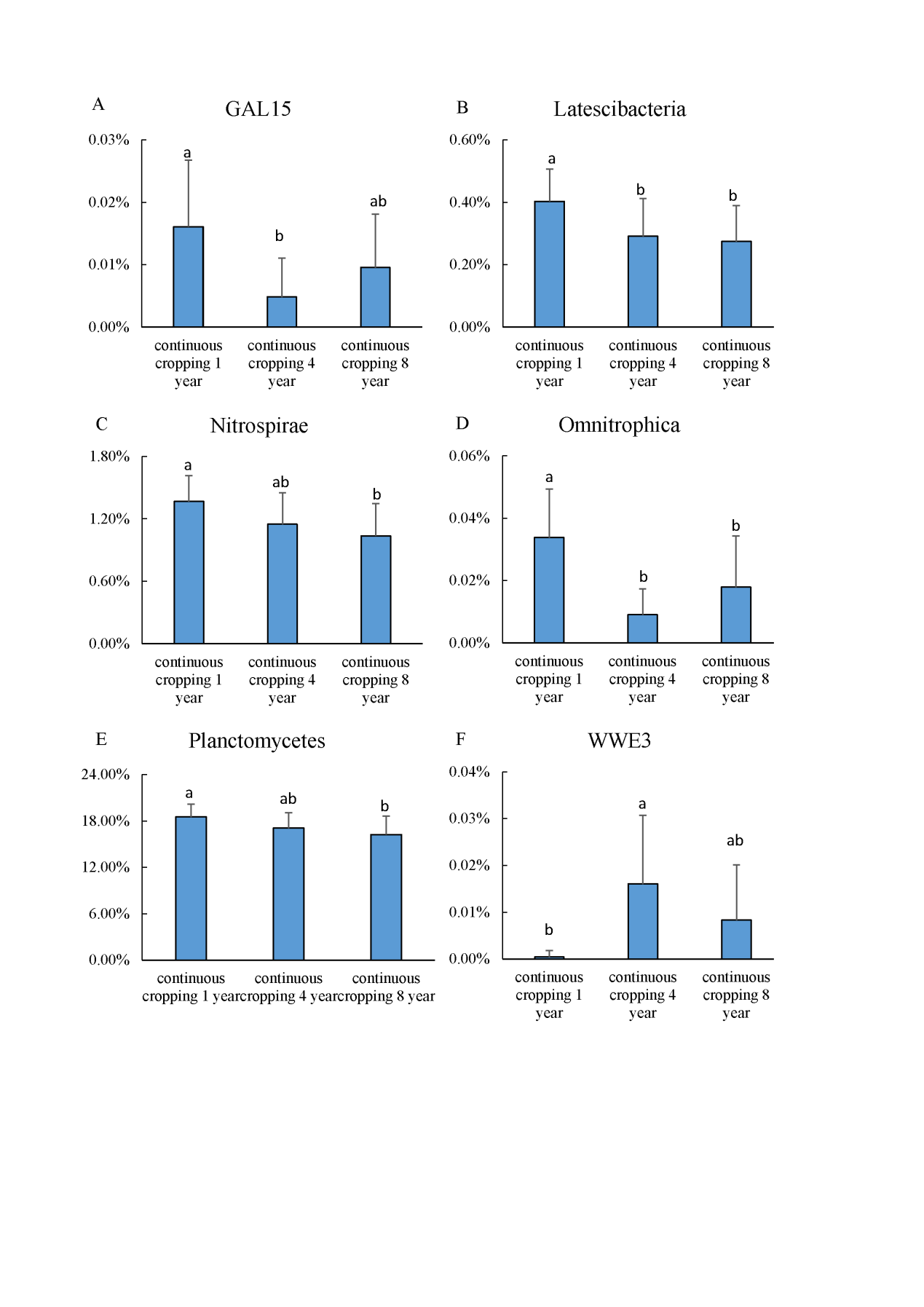


**Supplementary Figure S2.** Prokaryotic phyla that significantly change throughout *Potentilla anserina* development. (**A**) Euryarchaeota, (**B**) Acidobacteria, (**C**) Actinobacteria, (**D**) Armatimonadetes, (**E**) BRC1, (**F**) Bacteroidetes, (**G**) Chloroflexi, (**H**) Fibrobacteres, (**I**) Latescibacteria, (**J**) Parcubacteria, (**K**) Saccharibacteria, and (**L**) Verrucomicrobia. The bars with different letters are significantly different (analysis of variance Tukey post-hoc p < 0.05) from one another. Graphs show mean ± standard error.

**
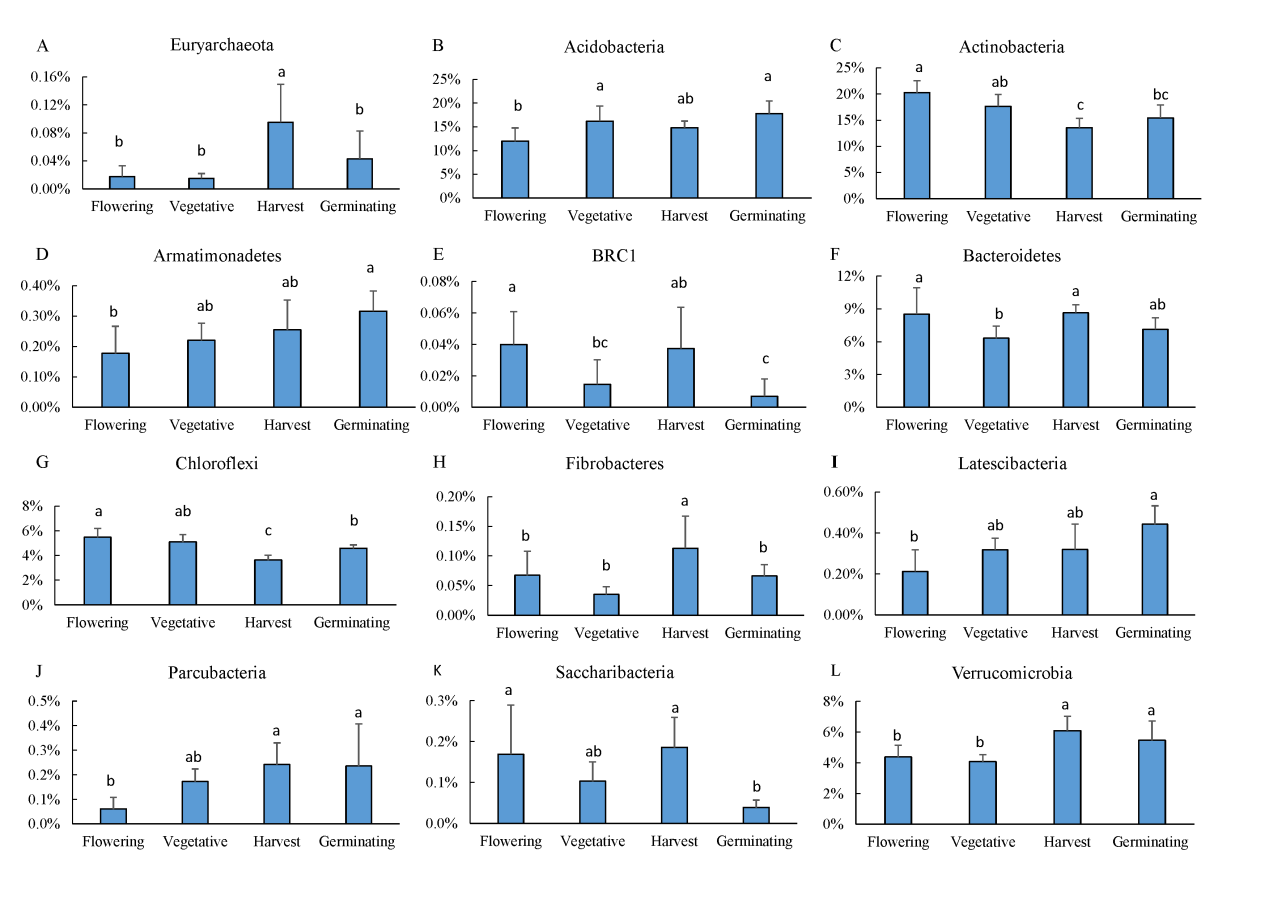
**

**Supplementary Table S1**. Observed species, Chao1, Shannon, Simpson, and Good’s_coverage diversity of *Potentilla anserina* rhizosphere prokaryotes.

| Samples No. | Observed_species | Chao1 | Shannon | Simpson | Good’s_coverage |
| --- | --- | --- | --- | --- | --- |
| SJ1 | 2469 | 2686.2150 | 10.2451 | 0.9984 | 0.9797 |
| SJ2 | 2361 | 2618.4274 | 10.1497 | 0.9983 | 0.9778 |
| SJ3 | 2340 | 2607.3488 | 10.1022 | 0.9981 | 0.9727 |
| TJ1 | 2374 | 2614.7580 | 10.1913 | 0.9982 | 0.9769 |
| TJ2 | 2219 | 2528.3750 | 10.0229 | 0.9980 | 0.9755 |
| TJ3 | 2270 | 2535.1545 | 10.0836 | 0.9981 | 0.9749 |
| KJ1 | 2308 | 2543.6244 | 10.2090 | 0.9985 | 0.9768 |
| KJ2 | 2124 | 2439.5655 | 9.9417 | 0.9981 | 0.9741 |
| KJ3 | 2373 | 2591.5878 | 10.4000 | 0.9987 | 0.9740 |
| SS1 | 2418 | 2633.5540 | 10.3271 | 0.9985 | 0.9788 |
| SS2 | 2467 | 2717.3846 | 10.2192 | 0.9982 | 0.9781 |
| SS3 | 2476 | 2700.9618 | 10.2565 | 0.9983 | 0.9790 |
| TS1 | 2444 | 2721.7434 | 10.1860 | 0.9982 | 0.9792 |
| TS2 | 2458 | 2670.3737 | 10.1977 | 0.9981 | 0.9810 |
| TS3 | 2441 | 2678.0241 | 10.1657 | 0.9981 | 0.9792 |
| KS1 | 2397 | 2624.0201 | 10.1844 | 0.9982 | 0.9805 |
| KS2 | 2426 | 2683.0855 | 10.1786 | 0.9982 | 0.9773 |
| KS3 | 2374 | 2620.8534 | 10.1670 | 0.9982 | 0.9788 |
| SN1 | 2473 | 2694.5040 | 10.3039 | 0.9984 | 0.9791 |
| SN2 | 2392 | 2675.5956 | 10.2153 | 0.9983 | 0.9754 |
| SN3 | 2422 | 2646.2966 | 10.2704 | 0.9984 | 0.9793 |
| TN1 | 2532 | 2751.8262 | 10.3283 | 0.9984 | 0.9798 |
| TN2 | 2436 | 2689.3238 | 10.2685 | 0.9984 | 0.9784 |
| TN3 | 2508 | 2682.1425 | 10.2845 | 0.9983 | 0.9813 |
| KN1 | 2417 | 2684.7236 | 10.1027 | 0.9980 | 0.9781 |
| KN2 | 2257 | 2579.3590 | 10.0936 | 0.9982 | 0.9770 |
| KN3 | 2519 | 2709.2567 | 10.3140 | 0.9984 | 0.9800 |
| SA1 | 2290 | 2587.1372 | 10.1977 | 0.9984 | 0.9757 |
| SA2 | 2344 | 2578.0990 | 10.2241 | 0.9984 | 0.9767 |
| SA3 | 2311 | 2559.0765 | 10.2183 | 0.9984 | 0.9765 |
| TA1 | 2401 | 2597.5393 | 10.3067 | 0.9984 | 0.9789 |
| TA2 | 2416 | 2644.1965 | 10.2458 | 0.9982 | 0.9806 |
| TA3 | 2396 | 2648.2927 | 10.3007 | 0.9985 | 0.9757 |
| KA1 | 2474 | 2694.7023 | 10.3064 | 0.9984 | 0.9779 |
| KA2 | 2250 | 2578.2869 | 10.1189 | 0.9982 | 0.9706 |
| KA3 | 2490 | 2731.7407 | 10.2864 | 0.9984 | 0.9782 |

Sample No.: S-Sitan village, T-Tuergan village, K-Kesuer village, J- June: flowering, S- [September](javascript:;): vegetative, N- November: harvest, A-April: germinating

**Supplementary Table S2.** Correlations between the measured environmental variables and the top 15 genera with the highest relative abundances within rhizosphere prokaryotes.

| Classification | Correlations with environmental variable | | | | | | | | | |
| --- | --- | --- | --- | --- | --- | --- | --- | --- | --- | --- |
|  |  | TN | TK | TP | AN | AK | AP | M | T | P |
| RB41 | *r* | 0.049 | -0.024 | -0.065 | -0.021 | 0.151 | -0.174 | -0.360^*^ | -0.225 | -0.029 |
|  | *P* | 0.778 | 0.891 | 0.708 | 0.905 | 0.380 | 0.309 | 0.031 | 0.187 | 0.868 |
| Sphingomonas | *r* | 0.015 | -0.283 | -0.450^**^ | -0.433^**^ | -0.521^**^ | -0.653^**^ | -0.330^*^ | -0.529^**^ | 0.435^**^ |
|  | *P* | 0.930 | 0.094 | 0.006 | 0.008 | 0.001 | 0.000 | 0.049 | 0.001 | 0.008 |
| Pir4 lineage | *r* | 0.017 | 0.022 | 0.123 | 0.153 | 0.116 | 0.308 | 0.184 | 0.182 | -0.029 |
|  | *P* | 0.922 | 0.899 | 0.474 | 0.374 | 0.499 | 0.067 | 0.282 | 0.289 | 0.868 |
| Pirellula | *r* | -0.047 | -0.024 | 0.063 | 0.035 | 0.165 | 0.300 | 0.006 | 0.100 | -0.045 |
|  | *P* | 0.784 | 0.892 | 0.715 | 0.840 | 0.335 | 0.075 | 0.971 | 0.560 | 0.792 |
| Blastococcus | *r* | -0.295 | -0.188 | -0.118 | -0.114 | 0.091 | 0.191 | -0.002 | 0.160 | 0.062 |
|  | *P* | 0.081 | 0.273 | 0.492 | 0.507 | 0.599 | 0.264 | 0.991 | 0.351 | 0.719 |
| Nocardioides | *r* | -0.242 | 0.212 | 0.116 | 0.051 | 0.267 | 0.461^**^ | 0.497^**^ | 0.811^**^ | -0.139 |
|  | *P* | 0.156 | 0.215 | 0.499 | 0.767 | 0.115 | 0.005 | 0.002 | 0.000 | 0.420 |
| Pseudarthrobacter | *r* | -0.450^**^ | -0.241 | -0.350^*^ | -0.359^*^ | -0.075 | -0.084 | 0.123 | 0.112 | 0.170 |
|  | *P* | 0.006 | 0.158 | 0.036 | 0.031 | 0.665 | 0.627 | 0.473 | 0.514 | 0.322 |
| Chthoniobacter | *r* | 0.039 | -0.016 | -0.360^*^ | -0.384^*^ | -0.336^*^ | -0.534^**^ | -0.187 | -0.416^*^ | 0.450^**^ |
|  | *P* | 0.820 | 0.927 | 0.031 | 0.021 | 0.045 | 0.001 | 0.276 | 0.012 | 0.006 |
| Lysobacter | *r* | -0.117 | -0.087 | -0.177 | -0.165 | -0.313 | -0.133 | 0.327 | 0.184 | 0.158 |
|  | *P* | 0.495 | 0.615 | 0.302 | 0.337 | 0.063 | 0.438 | 0.052 | 0.282 | 0.358 |
| Planctomyces | *r* | -0.069 | 0.046 | 0.138 | 0.083 | 0.092 | 0.270 | 0.477^**^ | 0.352^*^ | 0.098 |
|  | *P* | 0.690 | 0.791 | 0.420 | 0.630 | 0.592 | 0.112 | 0.003 | 0.035 | 0.569 |
| Opitutus | *r* | 0.033 | -0.117 | -0.071 | -0.066 | -0.226 | -0.256 | -0.143 | -0.536^**^ | -0.124 |
|  | *P* | 0.850 | 0.498 | 0.681 | 0.703 | 0.185 | 0.131 | 0.405 | 0.001 | 0.470 |
| Iamia | *r* | -0.200 | 0.468^**^ | 0.298 | 0.172 | 0.338^*^ | 0.473^**^ | 0.663^**^ | 0.823^**^ | -0.067 |
|  | *P* | 0.243 | 0.004 | 0.078 | 0.317 | 0.044 | 0.004 | 0.000 | 0.000 | 0.698 |
| AKYG587 | *r* | 0.511^**^ | -0.025 | 0.323 | 0.316 | 0.301 | 0.061 | -0.434^**^ | -0.368^*^ | -0.268 |
|  | *P* | 0.001 | 0.883 | 0.055 | 0.061 | 0.075 | 0.725 | 0.008 | 0.027 | 0.114 |
| Solirubrobacter | *r* | -0.203 | -0.036 | -0.065 | -0.108 | 0.056 | 0.175 | 0.117 | 0.297 | 0.160 |
|  | *P* | 0.235 | 0.835 | 0.706 | 0.530 | 0.745 | 0.308 | 0.498 | 0.079 | 0.351 |
| Arenimonas | *r* | -0.161 | -0.167 | -0.447^**^ | -0.357^*^ | -0.338^*^ | -0.319 | -0.125 | -0.129 | -0.017 |
|  | *P* | 0.349 | 0.330 | 0.006 | 0.033 | 0.044 | 0.058 | 0.468 | 0.453 | 0.923 |

The Spearman’s rank correlation coefficient (*r*) and significance of correlations (*P*) are shown. Correlations where *P* < 0.05 were considered to be significant. TN: total nitrogen, TK: total potassium, TP: total phosphorus, AN: available nitrogen, AK: available potassium, AP: available phosphorus, M: moisture, T: temperature, and P: accumulated precipitation 30 days before sampling time. * *P* < 0.05 and ** *P* < 0.01.

**Supplementary Table S3.** Environmental conditions of the soils from three agricultural fields in Huangyuan County.

| Sample No. | Date | Temperature | P | Moisture |
| --- | --- | --- | --- | --- |
|  |  | （℃） | (mm) | （%） |
| SJ1 | 2018.6.17 | 11.4 | 42.8 | 10.14 |
| SJ2 |  |  |  | 12.17 |
| SJ3 |  |  |  | 9.70 |
| TJ1 |  |  |  | 11.90 |
| TJ2 |  |  |  | 13.67 |
| TJ3 |  |  |  | 10.63 |
| KJ1 |  |  |  | 15.46 |
| KJ2 |  |  |  | 9.88 |
| KJ3 |  |  |  | 6.68 |
| SS1 | 2018.9.8 | 10.4 | 90.3 | 2.86 |
| SS2 |  |  |  | 2.43 |
| SS3 |  |  |  | 2.74 |
| TS1 |  |  |  | 1.72 |
| TS2 |  |  |  | 1.96 |
| TS3 |  |  |  | 1.89 |
| KS1 |  |  |  | 1.72 |
| KS2 |  |  |  | 1.77 |
| KS3 |  |  |  | 1.78 |
| SN1 | 2018.11.10 | -4.1 | 43.8 | 2.13 |
| SN2 |  |  |  | 1.56 |
| SN3 |  |  |  | 1.87 |
| TN1 |  |  |  | 1.28 |
| TN2 |  |  |  | 1.14 |
| TN3 |  |  |  | 1.13 |
| KN1 |  |  |  | 1.15 |
| KN2 |  |  |  | 1.16 |
| KN3 |  |  |  | 1.06 |
| SA1 | 2019.4.4 | 2.2 | 1.1 | 0.02 |
| SA2 |  |  |  | 0.03 |
| SA3 |  |  |  | 0.02 |
| TA1 |  |  |  | 0.03 |
| TA2 |  |  |  | 0.03 |
| TA3 |  |  |  | 0.02 |
| KA1 |  |  |  | 0.04 |
| KA2 |  |  |  | 0.02 |
| KA3 |  |  |  | 0.02 |

Sample No.: S-Sitan village, T-Tuergan village, K-Kesuer village, J- June: flowering, S- September: vegetative, N- November: harvest, A-April: germinating; P: accumulated precipitation 30 days before sampling time.

**Supplementary Table S4.** Environmental variables of *Potentilla anserina* rhizosphere soil.

| Sample No. | TN（g/kg） | TK（g/kg） | TP（g/kg） | AN（g/kg） | AK（g/kg） | AP（g/kg） |
| --- | --- | --- | --- | --- | --- | --- |
| KA1 | 1.4 | 18.4 | 0.728 | 0.123 | 0.072 | 0.021 |
| KA2 | 1.6 | 19.2 | 0.793 | 0.165 | 0.160 | 0.022 |
| KA3 | 1.8 | 18.8 | 0.902 | 0.151 | 0.104 | 0.027 |
| KJ1 | 1.7 | 19.4 | 0.839 | 0.222 | 0.146 | 0.027 |
| KJ2 | 1.5 | 19.4 | 0.873 | 0.130 | 0.104 | 0.024 |
| KJ3 | 1.8 | 19.7 | 0.967 | 0.166 | 0.191 | 0.031 |
| KN1 | 2.1 | 18.7 | 0.742 | 0.152 | 0.073 | 0.007 |
| KN2 | 1.9 | 18.9 | 0.820 | 0.154 | 0.094 | 0.012 |
| KN3 | 2.4 | 18.8 | 0.775 | 0.188 | 0.081 | 0.015 |
| KS1 | 1.2 | 19.4 | 0.774 | 0.139 | 0.118 | 0.012 |
| KS2 | 2.0 | 19.2 | 0.760 | 0.149 | 0.105 | 0.011 |
| KS3 | 2.0 | 19.0 | 0.794 | 0.131 | 0.118 | 0.013 |
| SA1 | 2.6 | 18.3 | 0.809 | 0.239 | 0.067 | 0.022 |
| SA2 | 2.7 | 18.5 | 0.905 | 0.199 | 0.185 | 0.026 |
| SA3 | 2.8 | 18.8 | 1.073 | 0.188 | 0.259 | 0.045 |
| SJ1 | 2.8 | 19.2 | 1.062 | 0.278 | 0.286 | 0.039 |
| SJ2 | 2.6 | 18.7 | 0.940 | 0.399 | 0.120 | 0.033 |
| SJ3 | 3.0 | 19.3 | 0.988 | 0.315 | 0.215 | 0.028 |
| SN1 | 2.2 | 19.0 | 0.907 | 0.196 | 0.094 | 0.019 |
| SN2 | 2.7 | 19.0 | 0.980 | 0.169 | 0.148 | 0.026 |
| SN3 | 2.9 | 18.7 | 0.854 | 0.186 | 0.102 | 0.016 |
| SS1 | 2.3 | 18.8 | 0.932 | 0.175 | 0.146 | 0.026 |
| SS2 | 2.8 | 19.1 | 0.983 | 0.202 | 0.103 | 0.033 |
| SS3 | 2.0 | 19.3 | 0.847 | 0.161 | 0.116 | 0.019 |
| TA1 | 2.0 | 19.8 | 0.872 | 0.171 | 0.199 | 0.030 |
| TA2 | 1.5 | 18.4 | 0.841 | 0.181 | 0.185 | 0.034 |
| TA3 | 1.6 | 19.4 | 0.857 | 0.195 | 0.330 | 0.030 |
| TJ1 | 1.4 | 18.9 | 0.805 | 0.189 | 0.121 | 0.023 |
| TJ2 | 1.7 | 19.3 | 0.924 | 0.374 | 0.253 | 0.054 |
| TJ3 | 1.1 | 18.6 | 0.812 | 0.128 | 0.114 | 0.036 |
| TN1 | 1.9 | 18.3 | 0.812 | 0.134 | 0.109 | 0.018 |
| TN2 | 1.6 | 17.8 | 0.741 | 0.084 | 0.087 | 0.013 |
| TN3 | 1.8 | 18.5 | 0.815 | 0.142 | 0.169 | 0.019 |
| TS1 | 2.1 | 18.7 | 0.771 | 0.123 | 0.110 | 0.027 |
| TS2 | 1.3 | 18.8 | 0.839 | 0.105 | 0.148 | 0.021 |
| TS3 | 1.2 | 18.9 | 0.810 | 0.121 | 0.168 | 0.024 |

Sample No.: S-Sitan village, T-Tuergan village, K-Kesuer village, J- June: flowering, S- [September](javascript:;): vegetative, N- November: harvest, A-April: germinating;

TN: total nitrogen, TK: total potassium, TP: total phosphorus, AN: available nitrogen, AK: available potassium, AP: available phosphorus
